# HAPHPIPE: Haplotype Reconstruction and Phylodynamics for Deep Sequencing of Intrahost Viral Populations

**DOI:** 10.1093/molbev/msaa315

**Published:** 2020-12-26

**Authors:** Matthew L Bendall, Keylie M Gibson, Margaret C Steiner, Uzma Rentia, Marcos Pérez-Losada, Keith A Crandall

**Affiliations:** 1 Computational Biology Institute, Milken Institute School of Public Health, The George Washington University, Washington, DC, USA; 2 Department of Biostatistics and Bioinformatics, Milken Institute School of Public Health, The George Washington University, Washington, DC, USA; 3 CIBIO-InBIO, Centro de Investigação em Biodiversidade e Recursos Genéticos, Universidade do Porto, Vairão, Portugal

**Keywords:** molecular epidemiology, phylodynamics, HIV, transmission cluster, bioinformatics

## Abstract

Deep sequencing of viral populations using next-generation sequencing (NGS) offers opportunities to understand and investigate evolution, transmission dynamics, and population genetics. Currently, the standard practice for processing NGS data to study viral populations is to summarize all the observed sequences from a sample as a single consensus sequence, thus discarding valuable information about the intrahost viral molecular epidemiology. Furthermore, existing analytical pipelines may only analyze genomic regions involved in drug resistance, thus are not suited for full viral genome analysis. Here, we present HAPHPIPE, a HAplotype and PHylodynamics PIPEline for genome-wide assembly of viral consensus sequences and haplotypes. The HAPHPIPE protocol includes modules for quality trimming, error correction, de novo assembly, alignment, and haplotype reconstruction. The resulting consensus sequences, haplotypes, and alignments can be further analyzed using a variety of phylogenetic and population genetic software. HAPHPIPE is designed to provide users with a single pipeline to rapidly analyze sequences from viral populations generated from NGS platforms and provide quality output properly formatted for downstream evolutionary analyses.

## Introduction

Phylogenetics and phylodynamics (see review Volz et al. 2013) have played a key role across a diversity of aspects for understanding viral evolution and population dynamics, including assessing agents of infection (e.g., the current COVID-19 pandemic; Andersen et al. 2020; Wu et al. 2020), intervention strategies (e.g., Ebola outbreaks; Dellicour et al. 2018), elucidating the origin of epidemics (e.g., HIV; Gao et al. 1999), and determining how those characteristics relate to host phenotypic data (Volz et al. 2013; du Plessis and Stadler 2015). Additionally, phylogenetic inference has provided critical insights into HIV biology in particular, from quantifying fitness costs of drug resistance (Kühnert et al. 2018) to identifying transmission networks (Ragonnet-Cronin et al. 2018)—even providing robust legal evidence in criminal trial cases (Metzker et al. 2002). Although ideally viral phylodynamics is investigated using whole-genome sequence data, in many cases and certainly for surveillance efforts, whole-genome sequencing is too costly or cumbersome and does not allow for dense population sampling of viral populations. Population focused transmission cases and drug resistance surveillance require denser sampling compared with the more broadly applied phylogenetic studies. Historically, such sampling was achieved by diluting viral population samples from infected individuals to a few viral particles and then using polymerase chain reaction (PCR) to amplify particular gene fragments to use in phylogenetics. Ideally, multiple independent dilutions and subsequent rounds of PCR would occur per infected individual to achieve population-based sampling within and among infected individuals. The resulting sequences from Sanger sequencing would be treated as “haplotypes” (sequence variants) for downstream traditional phylogenetic analyses (alignment, model selection, phylogeny estimation, etc.). Thus, phylodynamic studies can take advantage of commercial data sets focused on drug resistance screening efforts (Pérez-Losada et al. 2017).

**Fig. 1.**
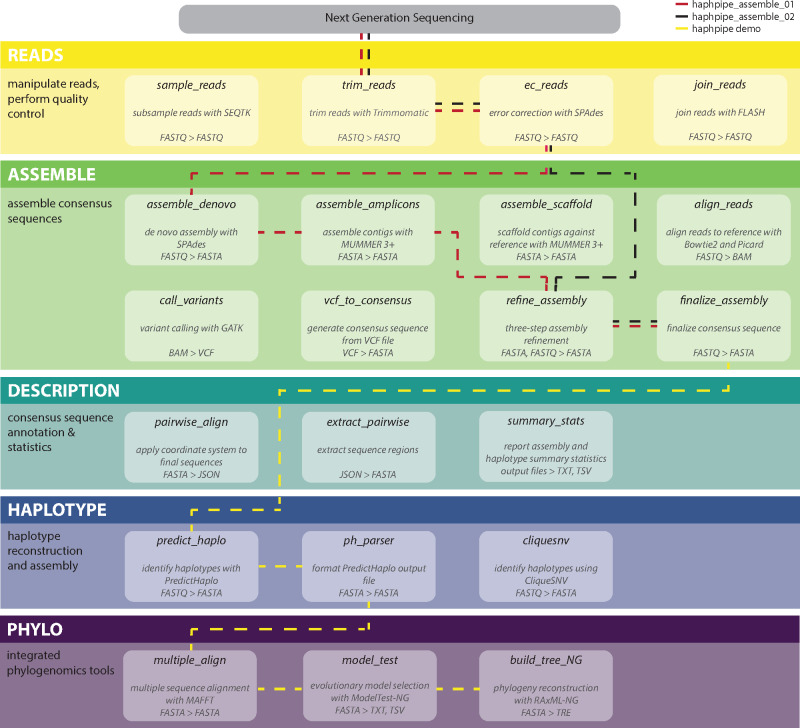
HAPHPIPE schematic. Each color represents a different stage of the analysis pipeline, with the corresponding modules located within each stage. Two sample pipelines, haphpipe_assemble_01 and haphpipe_assemble_02, are shown in red- and black-dashed lines, respectively. The demo pipeline uses haphpipe_assemble_02 and the remaining steps are shown in yellow-dashed lines. Input and output file formats are listed for each module.

With the advent of next-generation sequencing (NGS), a much larger volume of data can be collected for phylodynamic studies requiring different bioinformatic approaches, especially for early data treatment in the bioinformatics pipeline (Pérez-Losada et al. 2020). With either shotgun sequencing reads of viral populations or targeted amplicon sequencing (Bybee et al. 2011) of genes of interest (typically protease [*PR*], reverse transcriptase [*RT*], and integrase [*int*] for HIV drug resistance studies, but also envelope [*env*] for transmission studies), NGS data require additional steps to convert these short raw sequence reads into haplotypes for downstream analyses with tested phylogenetic and population genetic software packages (e.g., Gibson et al. 2020a). Thus, we have created HAPHPIPE—a HAplotype reconstruction and PHylodynamics PIPEline for genome-wide assembly of viral consensus sequences and haplotypes from next-generation viral sequence data (fig. 1). In this protocol, we provide detailed instructions for executing all available HAPHPIPE stages (set up in modular format) and present two example pipelines we have included with this software. We assume some familiarity with NGS platforms and analyses (reviewed in Levy and Myers 2016). Additionally, we assume some familiarity with bash/command line to execute HAPHPIPE. In addition to the included phylogenetics stages, output files from HAPHPIPE can be easily used as inputs for other phylogenetic and phylodynamic software tools such as BEAST2 (Bouckaert et al. 2014), HIV-TRACE (Kosakovsky Pond et al. 2018), and DnaSP (Rozas et al. 2017), to name a few.

HAPHPIPE has been thoroughly compared with similar viral assembly tools and has been validated against two commonly used programs: HyDRA and Geneious (Gibson et al. 2020b). Both of these programs use graphical user interfaces, with HyDRA being free and web-based, and Geneious being commercial. Briefly, we found that HAPHPIPE performed significantly better than HyDRA, comparable to or better than Geneious (depending on viral gene region) and could accommodate larger data sets with a quicker turnaround time. Furthermore, unlike some programs including HyDRA which are virus-specific, any virus can be analyzed within HAPHPIPE; we have validated HAPHPIPE on HIV, HCV, and SARS-CoV-2 data. Although HAPHPIPE is terminal based, we intend for our User Guide to be an extension of our tool and provide ample information for any level user to analyze their own data with HAPHPIPE.

## Protocol

HAPHPIPE is constructed in a modular fashion, in which the different stages are categorized into five main sections: quality control, assembling sequences, haplotype assembly, description, and phylogenetics. We envision HAPHPIPE to be accessible and helpful to a range of users. Beginning users may rely heavily on the example pipelines provided, whereas experienced users with expertise in NGS analysis may design new pipelines using the modules provided and/or swap in/out alternative software at particular steps in the workflow. HAPHPIPE is also extensible; users who are familiar with python can easily create new stages or modules that take advantage of HAPHPIPE utility functions. We strive to follow best practices in developing our pipelines and protocol including availability of source-code, software indexing, documenting, and management of source code, and providing test libraries, sample data, and data set repositories (Leprevost et al. 2014).

HAPHPIPE was conceived to work in Linux and Mac OS X operating systems, however it can be run on a PC using a virtual machine (see User Guide at https://gwcbi.github.io/haphpipe_docs/install/#windows-users for more information). HAPHPIPE depends on more than a dozen different programs, each of which may itself depend on other programs and libraries. Installing everything separately is complex and daunting, so we recommend the use of the popular package manager “Conda” with the channel “Bioconda” to install HAPHPIPE (Dale et al. 2018). All the packages used in HAPHPIPE are widely used, open source, and have been previously validated and available in Bioconda. The User Guide (https://gwcbi.github.io/haphpipe_docs/install/#haphpipe-installation-instructions) describes how to download and install Bioconda and then how to use it to install HAPHPIPE (i.e., with a single command: conda create -n haphpipe haphpipe). The User Guide (https://gwcbi.github.io/haphpipe_docs/install/#haphpipe-installation-instructions) details the acquisition and installation of one program, GATK (McKenna et al. 2010), that is not handled by Bioconda, and finally the acquisition and installation of HAPHPIPE itself. Upon completion of the installation, you can test it to ensure the repository has been installed completely and correctly by running haphpipe -h. Once HAPHPIPE is installed and performing correctly, there is no need to install it again; simply activate the conda environment when needed by executing conda activate haphpipe. At any point, the -h option that follows any HAPHPIPE modules will output a help message that provides a description of the module and the desired input(s) and output(s).

Furthermore, for the haplotype stage (hp_predict_haplo and hp_ph_parser), users are required to install PredictHaplo (Prabhakaran et al. 2014) on their own, as there are system-dependent variables within the installation process. More information and documentation on how we implemented PredictHaplo in our system can be found at https://gwcbi.github.io/haphpipe_docs/install/#predicthaplo-installation-instructions.

### Starting Point for HAPHPIPE

**Fig. 2.**
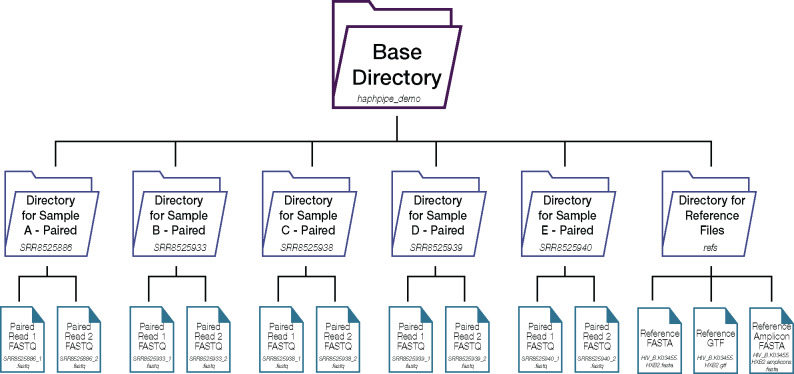
Example starting directory structure. The base directory should contain one subdirectory for each sample (with a unique and descriptive name containing sequence data and a separate subdirectory for the file(s) of viral reference genome(s) and/or genes). The example here shows the structure for the demo data, with each sample used in the demo as an individual subdirectory housing two FASTQ files—one for read1 and another for read2 (these are paired read files). The reference subdirectory has three files that are used in the demo: a reference FASTA file (HIV_B.K03455.HXB2.fasta), an amplicon reference FASTA file (HIV_B.K03455.HXB2.amplicons.fasta), and a tab-delimited text file contain genomic structure information (HIV_B.K03455.HXB2.gtf). The structure of a subdirectory containing only single end reads would be the same, except there is only one FASTQ file per sample (instead of two show here for paired-end reads).

Prior to running HAPHPIPE on any NGS viral samples, we will first describe the recommended directory structure (see https://gwcbi.github.io/haphpipe_docs/install/for more detail) for running HAPHPIPE. A directory is synonymous with a folder on your computer, with folders containing other subfolders and files (fig. 2). The base, or main, directory should contain a single subdirectory for the file(s) of viral reference genome(s) and/or genes and one subdirectory for each sample (with a unique and descriptive name) containing sequence data. The starting point for each sample is a file or files of sequences in FASTQ format, where the file size depends on the size of the genome and the sequencing coverage. If coverage is somewhere between 20× and 100×, the average read is 250 bp, and the genome is 10 kb, there will be somewhere between 800 and 4,000 sequences in the FASTQ file. If the reads are paired-end with Illumina sequencing technology, then there will be two FASTQ files, each with between 400 and 2,000 reads. The purpose of HAPHPIPE is to assemble these reads into a single FASTA file (no sequencing quality scores) containing haplotype sequences, which often includes between one and ten sequences in the resulting FASTA file. Genome assembly may be assisted by the inclusion of a closely related genome sequence, that is, reference sequence, that has already been assembled, or assembly may be “de novo,” that is, without the assistance of any known genome sequences. Often a reference sequence may be preferred if one exists in high quality. We demonstrate both approaches below with example pipelines and demo data.

The reference directory (refs/) contains files of reference genomes for reference-based assembly (fig. 2). For this protocol, we demo HAPHPIPE using HIV-1 data, and for these examples, we use the HIV reference using three files: HIV_B.K03455.HXB2.fasta, HIV_B.K03455.HXB2.amplicons. fasta, and HIV_B.K03455.HXB2.gtf. All files are available in the User Guide (https://gwcbi.github.io/haphpipe_docs/install/#reference-files) and by direct download through the demo module (using the –refsonly option). The full HIV-1 HXB2 genome reference sequence is located in FASTA format (Pearson and Lipman 1988) in HIV_B.K03455.HXB2.fasta. This file is used in the first example pipeline (described below), where we are using it as the reference genome for assembly. There are three reference genes (*PRRT*, *int*, and *gp120*) located in FASTA format in the HIV_B.K03455.HXB2.amplicons.fasta file, and we use this file in the second example pipeline. Finally, the GTF file (a gene transfer format file) (Zerbino et al. 2017; Lopez et al. 2019) is a tab-delimited text file that holds information about the gene structure and primer binding sites. In our case, for the demo data and the example pipelines, we use the starting and stopping base numbers (based on the numbering scheme for HXB2; see information regarding numbering scheme below) for the three amplicons of interest. Details on these HIV reference files can also be found on the User Guide (https://gwcbi.github.io/haphpipe_docs/install/#reference-files).

### HAPHPIPE Workflow

Here, we detail all of the stages of the HAPHPIPE workflow, including quality control, sequence assembly, description, haplotype assembly, and phylogenetics (fig. 1). Additional options for each module are available by entering a command with the -h option in the command line (e.g., haphpipe ec_reads -h) as well as at https://gwcbi.github.io/haphpipe_docs/install/#quick-start. In HAPHPIPE, modules may be called either with the addition of “hp_” to the module name (e.g., hp_sample_reads) or by first calling HAPHPIPE (e.g., haphpipe sample_reads). For the purposes of this protocol, we will consistently use the latter. Furthermore, a complete list of the required input format(s) for and the file outputs from each module are maintained in a table at https://gwcbi.github.io/haphpipe_docs/inout/. HAPHPIPE currently contains five stages. Stage 1 involves cleaning the raw read sequences; Stage 2 involves assembling the clean read data to produce assembled genome sequences in FASTA format and generates a consensus sequence; Stage 3 involves generating haplotypes; Stage 4 involves descriptive options, such as annotating the sequences, extracting gene regions, and calculating summary statistics; and Stage 5 involves phylogenetic steps, including multiple sequence alignment, best-fit evolutionary model testing and constructing a phylogenetic tree. Below, we address each stage and explain each module within the stage. We also split the stages into basic (Stages 1 and 2) and advanced (Stages 3, 4, and 5) concepts.

#### Basic Concepts

##### Stage 1: Quality Control

The first step in NGS data analysis is quality control. Modern NGS platforms (i.e., Illumina) output raw sequencing data in compressed FASTQ format (Cock et al. 2010), which contains the base calls and a quality score for each base, reflecting the confidence that the base was called correctly. For paired-end sequencing runs, the data are usually split between two files, one for each read. The error model and biases of Illumina sequencing are well understood (Schirmer et al. 2015), and several quality control steps may be needed. For example, read trimming is often used to remove adapter sequences from the reads and remove low-quality bases that occur toward the 3′-end of the read. Other modules for manipulating data at the “read” level are described below.

###### Read Trimming (trim_reads)

Reads frequently contain flanking regions of lower sequencing quality base pairs, which must be removed before continuing with further analysis. Additionally, a read may have an average quality score below a given threshold and, thus, removal of that read is desired. Furthermore, because NGS library preparation utilizes adapter sequences to bind the target DNA to the sequencing platform’s chemistry and/or to differentiate samples on a pooled sequencing run, adapters may remain present in the reads even after demultiplication steps. This adapter contamination can be handled by trimming the reads of these adapter sequences as well. In HAPHPIPE, the hp_trim_reads module uses Trimmomatic v. 0.39 (Bolger et al. 2014) to perform quality trimming on reads. This module takes as input raw reads as FASTQ files, compressed or not, and outputs trimmed FASTQ files. Example to execute: haphpipe trim_reads –fq1 SRR8525886_1.fastq –fq2 SRR8525886_2.fastq.

###### Error Correction (ec_reads)

The goal of error correction is to locate and remove errors in sequence data introduced into the sequence reads by the sequencing platform. Error correction is recommended for some downstream applications, including de novo assembly. On the other hand, error correction may not be appropriate for other applications such as population-level variant calling. Error correction is performed in HAPHPIPE through SPAdes v. 3.13.1 (Bankevich et al. 2012). To run error correction, input FASTQ files, which may be trimmed beforehand. Error correction can also be performed during de novo assembly in hp_assemble_denovo. Example to execute: haphpipe ec_reads –fq1 trimmed_1.fastq –fq2 trimmed_2.fastq.

###### Joining (join_reads)

Joining (or merging) read pairs is an optional step for paired-end sequencing designs. When the fragment length is less than the combined read length for both pairs, the 3′-ends will overlap and may be joined to form a single long read. For example, if a 2 × 300 kit is used to sequence 500-bp fragments, read pairs can be joined for an effective 500-bp read length with 100-bp overlap. Longer reads may improve genome assembly and haplotype reconstruction. FLASh v. 1.2.11 (Magoč and Salzberg 2011) is a method to merge paired-end reads into a single, longer sequence by identifying overlapping regions, which precludes common issues in de novo assembly arising from short read lengths. This step is implemented in HAPHPIPE in the hp_join_reads module, which outputs a single joined FASTQ file. Example to execute: haphpipe join_reads –fq1 trimmed_1.fastq –fq2 trimmed_2.fastq.

###### Subsampling (sample_reads)

Due to the large size of NGS data sets, it may be desirable to randomly subsample a data set for testing and computational efficiency. The HAPHPIPE module hp_sample_reads allows users to subsample a given number or fraction of reads from the file with seqtk v. 1.3 (https://github.com/lh3/seqtk). To do so, simply input the FASTQ files (raw, cleaned, or corrected—this is dependent upon your intentions) and specify the number or fraction of reads to sample. Example to execute: haphpipe sample_reads –fq1 SRR8525886_1.fastq –fq2 SRR8525886_2.fastq –nreads 1,000 –seed 1234.

##### Stage 2: Assembling Sequences

Many assembly strategies are designed for the assembly of a single genome from NGS data (Nagarajan and Pop 2013; Simpson and Pop 2015). However, in our case, we are interested in identifying variants and assembling multiple variant genes or genomes—targeted assembly (Warren and Holt 2011). The HAPHPIPE protocol implements two different strategies for targeted sequence assembly, namely: reference-based and de novo assembly. Reference-based assembly involves aligning (or mapping) reads to a reference sequence then generating a new sequence based on the consensus of the alignments. Compared with de novo assembly, reference-based assembly is computationally efficient, has lower memory requirements, and requires reduced sequencing depth. However, this approach is dependent on the availability of a closely related reference sequence, and the chosen reference will tend to bias the final assembly. In contrast, de novo sequence assembly requires no prior knowledge of the genome. In this approach, the full sequence is reconstructed by identifying overlaps among sequence reads. The amount of RAM needed for de novo assembly can be prohibitive for large genomes or metagenomes but is feasible (using a moderate workstation) for small genomes such as HIV-1.

De novo assembly in HAPHPIPE begins with the hp_assemble_denovo module, which uses the SPAdes (Bankevich et al. 2012) assembler to assemble short sequencing reads into longer sequences called contigs. The contigs produced may not span the entire target region or be in reverse orientation compared with the reference, so we perform a scaffolding step to orient and merge contigs. HAPHPIPE provides two options for scaffolding: the hp_assemble_amplicons module creates one sequence per amplicon region, whereas hp_assemble_scaffold creates one sequence with uncovered regions filled in with “Ns.”

Reference-based assembly begins with the hp_assemble_reads module, which aligns all the reads to the provided reference using Bowtie2 (Langmead and Salzberg 2012). Next, differences from the reference are called using GATK (McKenna et al. 2010) in the hp_call_variants module, and an updated consensus sequence is generated using hp_vcf_to_consensus. These three modules can be run sequentially using the hp_refine_assembly module. The hp_refine_assembly module also enables iterative refinement of the consensus sequence by using the consensus from one round as the reference for the next.

###### De Novo Assembly (assemble_denovo)

The hp_assemble_denovo module is implemented as a convenience wrapper around the SPAdes assembler. The SPAdes assembler (Bankevich et al. 2012) uses a De Bruijn graph assembly approach (see Nagarajan and Pop [2013] for more information on De Bruijn graph assembly). Inputs for the hp_assemble_denovo module are the read files in FASTQ format. With default settings, this module will automatically run error correction (this may be turned off using the –no_error_correction option). The assembled contigs are output in FASTA format. Example to execute: haphpipe assemble_denovo –fq1 corrected_1.fastq –fq2 corrected_2.fastq –outdir denovo_assembly –no_error_correction TRUE.

###### Amplicon Assembly (assemble_amplicons)

After de novo assembly, contigs are scaffolded with MUMMER 3+ (Kurtz et al. 2004) to form a consensus sequence in the hp_assemble_amplicons module. To assemble contigs using both a reference sequence and amplicon regions, input the contigs and reference sequence in FASTA format and the amplicon regions in GTF format. Example to execute: haphpipe assemble_amplicons –contigs_fa denovo_contigs.fa –ref_fa HIV_B.K03455.HXB2. fasta –ref_gtf HIV_B.K03455.HXB2.gtf.

###### Scaffolding (assemble_scaffold)

In this module, hp_assemble_scaffold, contigs are scaffolded, again with MUMMER 3+, against a reference sequence. Simply input the contigs and reference sequence (both in FASTA format) into HAPHPIPE, as shown below. Example to execute: haphpipe assemble_scaffold –contigs_fa denovo_contigs.fa –ref_fa HIV_B.K03455. HXB2.fasta.

###### Alignment to Reference (align_reads)

Reads may also be mapped to a reference sequence, instead of running de novo assembly. This concept is often seen in HIV studies, where if a host’s virus is known to be subtype B, then the sample’s reads are simply mapped against the HIV reference sequence HXB2 (Ratner et al. 1985). In this HAPHPIPE module hp_align_reads, alignment of the reads to the reference sequence is performed using Bowtie2 (Langmead and Salzberg 2012). The alignment files are then scanned for technical duplicates and indels are realigned using Picard (http://broadinstitute.github.io/picard/). As shown below, one can input both read files (in FASTQ format) and the reference file (in FASTA format). Example to execute: haphpipe align_reads –fq1 corrected_1.fastq –fq2 corrected _2.fastq –ref_fa HIV_B.K03455.HXB2.fasta.

###### Variant Calling (call_variants)

Variant calling identifies differences by nucleotide position between a reference sequence and the assembly. In the context of HAPHPIPE, the reference sequence can either be the sequence used to align the reads against (reference-based assembly) or the consensus final sequence from de novo assembly. The Variant Call Format (VCF) file (Danecek et al. 2011) produced by this stage represents intrasample diversity and contains the position of a variant within a sequence, the reference base, and alternative bases found along with a quality score for each allele. In HAPHPIPE, we perform variant calling with GATK v. 3.8 in the module hp_call_variants by inputting an alignment file (BAM format) and a reference sequence (FASTA format). Example to execute: haphpipe call_variants –aln_bam alignment.bam –ref_fa HIV_B.K03455.HXB2.fasta.

###### Consensus Sequence from Variants (vcf_to_consensus)

Alternatively, a VCF file containing information about variants in the sequencing data compared with a reference can then be used to generate a consensus sequence. In the hp_vcf_to_consensus module in HAPHPIPE, input the VCF file as shown. Example to execute: haphpipe vcf_to_consensus –vcf variants.vcf.

###### Refine Assembly (refine_assembly)

In the hp_refine_assembly module, corrected reads are first mapped either to a de novo assembly or reference sequence and variants are called. Then, in an iterative process, the reference is updated and the process repeats. The iteration ends when no improvement is made after an additional iteration. At this point, the refined reference sequence is outputted in FASTA format, which is then used to finalize the assembly (below). The user can specify the maximum number of steps within the refinement module, which represents the maximum times the reads will be mapped to the sequence and updated. If the refined sequence reaches a convergence, where the alignment rate and number of differences between the initial and new refined sequence do not improve, prior to the number of maximum steps, then the refinement module ends once this convergence is met. Likewise, if the refined sequence continues to be improved in each step but hits the maximum step, then the refined sequence at the last step is considered the final refined sequence. This option to include a maximum number of refinement steps is to allow users to specify how many steps are needed or requested based on available computational time or user desire. In our validation study, we found that often two or three refinement steps are efficient in creating a better, more representative consensus sequence (Eliseev et al. 2020). Example to execute: haphpipe refine_assembly –fq_1 corrected_1.fastq –fq2 corrected_2.fastq –ref_fa HIV_B.K03455.HXB2.fasta.

###### Finalize Assembly (finalize_assembly)

In the final HAPHPIPE assembly module, hp_finalize_assembly, the consensus sequence is finalized, reads are mapped to this consensus, and variants are called relative to the sample’s consensus sequence (final.fna). The outputs include final versions of the reference sequence, alignment, and variants (in FASTA, BAM, and VCF formats, respectively). Example to execute: haphpipe finalize_assembly –fq_1 corrected_1.fastq –fq2 corrected_2.fastq –ref_fa refined.fna.

#### Advanced Concepts

##### Stage 3: Haplotype assembly

With the aligned reads and variants, we can generate both a consensus sequence and haplotypes from the within sample variation. Often haplotypes are preferred for estimating transmission clusters (Gibson et al. 2020a), testing for associations between clinical variables (phenotypes) and genetic variation within individuals versus among individuals (Gibson et al. 2020a), and characterizing drug resistance variants (Johnson et al. 2008; Metzner et al. 2009; Simen et al. 2009; Li et al. 2011) (see Posada-Cespedes et al. [2017] for brief overview of advantages to using haplotypes). Thus, we have incorporated the common haplotype callers PredictHaplo (Prabhakaran et al. 2014) and CliqueSNV (Knyazev et al. 2018) as modules within HAPHPIPE. Viral haplotype reconstruction is an area of ongoing research. Alternative haplotype reconstruction programs, such as QuasiRecomb (Topfer et al. 2013), SAVAGE (Baaijens et al. 2017), ShoRAH (Zagordi et al. 2011), and others (see Posada-Cespedes et al. [2017] for review and Eliseev et al. [2020] for comparison of haplotype reconstruction algorithms) can be utilized outside of HAPHPIPE or advanced users can swap out an alternative haplotype caller for PredictHaplo or CliqueSNV. We have incorporated PredictHaplo and CliqueSNV because those perform best for viral diversity levels (Eliseev et al. 2020). Many of these haplotype reconstruction programs’ inputs are provided by the final output of HAPHPIPE—typically, the final consensus FASTA file, the raw, trimmed, and/or corrected reads, and/or the final BAM file can be used as input.

###### PredictHaplo (predict_haplo)

Once PredictHaplo is installed on your device, the required inputs for the HAPHPIPE module hp_predict_haplo are the paired-end FASTQ read files and a reference sequence FASTA file. The main outputs from this module are the longest global haplotype file (in PredictHaplo’s format) and the HTML file corresponding to the longest global haplotype file. By default, this module uses all the amplicons that are present in the reference sequence file. However, an interval text file can be supplied by the user if alternative intervals are desired. This file is suitable when a full genome is constructed but you are only interested in a particular interval (e.g., gene region, such as protease in HIV). Along with this module goes its complementary module entitled hp_ph_parser (see below), which generates a usable FASTA file from the output of this module. Example to execute: haphpipe predict_haplo corrected_1.fastq –fq2 corrected_2.fastq –ref_fa final.fna.

###### Parsing PredictHaplo Output (ph_parser)

PredictHaplo outputs a “.fas” file containing the reconstructed haplotypes, albeit this is not a true FASTA file. It contains a frequency for the reconstructed haplotype, quality scores, and confidence scores along with the haplotype sequence. Using the longest global haplotype output from the PredictHaplo module (hp_predict_haplo) as input for this module hp_ph_parser, the output is a correctly formatted FASTA file with each reconstructed sequence. Example to execute: haphpipe ph_parser PH01.best_1_864.fas.

###### CliqueSNV (clique_snv)

To generate haplotypes using CliqueSNV, you must first download the CliqueSNV JAR file (available at https://github.com/vtsyvina/CliqueSNV). Then, the required inputs to the HAPHPIPE module are FASTQ read file(s) (if using single read files, additionally use the –fqU option), a reference FASTA file, and the path to the directory containing the CliqueSNV JAR file (if not the current directory). The outputs of this module are one FASTA file containing reconstructed haplotypes and a TXT file containing CliqueSNV run information and results, as well as an additional TXT summary file with parsed results from this file (similar to the output of ph_parser), which provides the number of haplotypes reconstructed, the haplotype diversity estimate, and the nucleotide length of the haplotypes. Example to execute: haphpipe clique_snv –fq1 corrected_1.fastq –fq2 corrected_2.fastq –ref_fa final.fna –jardir ∼/Downloads.

##### Stage 4: Description

After processing the sequencing data, postanalyses steps often require specific formatting requirements, such as alignment of the sequences or extracting a particular gene region from all samples for phylogenetic analyses. HAPHPIPE provides a few modules to help with such data parsing and manipulations. Outputs from these modules are intended to be correctly formatted for input into different programs for phylodynamic studies or can be easily adapted by the user for further analyses, such as complying with program specific input requirements or exploring data (e.g., the BAM file) with SAMtools (Li et al. 2009) or genome browsers, such as the Integrative Genomics Viewer (IGV) (Robinson et al. 2011, 2017; Thorvaldsdóttir et al. 2013). Some programs, such as LDHat, PAML, and HyPhy, which analyze molecular epidemiology statistics, require modified FASTA files that can be edited by the user to satisfy the program’s requirements. DnaSP, a program that measures genetic diversity estimates, requires an aligned nucleotide FASTA file, which can be obtained from the modules below. Furthermore, the FASTA outputs can be directly input into the Stanford Database (https://hivdb.stanford.edu) to detect drug resistant mutations (DRMs) in HIV-1 sequences.

###### Coordinate Orientation (pairwise_align)

Because of their high mutation rates and high replication rates, retroviruses in general and HIV in particular, are prone to insertions and deletions (indels) in their genomes. Therefore, it was determined by the HIV community in 1998 that a designated numbering coordinate system was needed for the HIV-1 virus. As a result, the HIV-1 HXB2 sequence was introduced as to anchor such a system (Korber et al. 1998). HXB2 (Ratner et al. 1985) (GenBank accession number: K03455) clearly presents all the proteins with numbering for both the amino acid and nucleotide positions. This has provided a single numbering and reference for all HIV-1 studies, where the precise location of DNA or protein(s) of interest can be accurately reported. The concept of a numbering scheme to identify nucleotides and amino acids corresponding to genes or regions of interest also applies to other viruses, such as influenza (Burke and Smith 2014), polyomavirus BK (Luo et al. 2009), and hepatitis C (Kuiken and Simmonds 2009). In HAPHPIPE, the concept of this module hp_pairwise_align is to get final sequence(s) into the corresponding coordinate system to facilitate downstream analyses. An amino acid pairwise alignment is completed with the final sequence and the reference sequence with annotated gene regions. To run the coordinate orientation, the final sequence FASTA file, a reference sequence FASTA file, and a reference GTF file delimiting gene regions of interest are used as input. The output is a JSON (javascript object notation) file (Bray 2014), which is used as input for the following step (hp_extract_pairwise), in which the amplicons of interest are extracted. Example to execute: haphpipe pairwise_align –amplicons_fa final.fna –ref_fa HIV_B.K03455.HXB2.fasta –ref_gtf HIV_B.K03455.HXB2.gtf.

###### Extract Sequence Regions from Pairwise Alignment (extract_pairwise)

Phylogenetic analyses of viruses are often focused on individual genes for different biological reasons, for example, *PR* for drug resistance, *env* for transmission linkages, etc. (Rambaut et al. 2004; Castro-Nallar et al. 2012). Furthermore, viral sequences often undergo recombination and these recombination breakpoints tend to lie at gene boundaries and can also impact phylogenetic estimation (Posada and Crandall 2002; Posada et al. 2002). Therefore, the module hp_extract_pairwise extracts sequence regions from the pairwise alignment produced in the module above (hp_pairwise_align). For example, you may need this if you are primarily interested in the protease gene in HIV-1 from the polymerase (*pol)* alignment. The input is the JSON file from the previous step, and the output can be designated as an unaligned nucleotide FASTA file, an aligned nucleotide FASTA file, an amino acid FASTA file, an amplicon GTF file, or a tab-separated values (TSV) file. By default, a nucleotide FASTA file is the output and the regions of interest from the GTF file in the module above is used for the regions. However, a region can be designated by the name and starting and ending positions. Example to execute: haphpipe extract_pairwise –align_json pairwise_aligned.json –refreg HIV_B.K03455.HXB2:2085-5096.

###### Summary Statistics (summary_stats)

When working with and analyzing NGS data, it is often good practice to evaluate the number of reads throughout analysis steps. Furthermore, it is helpful to view a summary of the analysis for each sample and the statistics associated with that sample. Thus, we have incorporated a module to summarize and provide quick and simple data descriptions. The output of this module is a text file and its corresponding TSV files (for easy uploading into RStudio; RStudio Team 2015; Excel, or similar programs) that include for each individual sample: 1) the number of raw reads, 2) the number of cleaned reads, 3) alignment rate, 4) amplicon length, 5) amplicon read count, 6) amplicon coverage, 7) number of single nucleotide polymorphisms (SNPs) in the amplicon, and 8) Watterson’s theta measurement of genetic diversity. If multiple amplicons are included in the analyses, each amplicon is reported with its respective summary statistics. Theta is measured as the number of SNPs divided by the length of the amplicon. The input is a list of sample directories which contain the required files, which are: final_bt2.out (the final Bowtie2 output), trimmomatic_summary.out (the Trimmomatic summary file), final.fna (the final consensus file), final.vcf.gz (the final VCF file), and final.bam (the final BAM file). If using the sample pipelines below (haphpipe_assemble_01 and haphpipe_assemble_02), this list of sample directories will be $sampID/haphpipe_assemble_01 or $sampID/haphpipe_assemble_02, respectively, where $sampID is the name of the sample directory. Furthermore, if haplotypes were reconstructed using PredictHaplo (using the haplotypes modules hp_predict_haplo and hp_ph_parser), then the output summary files from these steps can be included in the output summary statistics text file. To include this information in the summary statistics, another list of directories which contain the files ph_summary.txt need to be included in the input (e.g., $sampID/haphpipe_assemble_01/PH01). Example to execute: haphpipe summary_stats –dir_list demo_sra_list.txt –ph_list demo_sra_ph_list.txt –ref_gtf HIV_B.K03455.HXB2.gtf.

##### Stage 5: Phylogenetics

A significant aspect of phylodynamics is estimating phylogenetic trees. Phylogenetics is the study of relationships and evolutionary history among taxa/sequences, which in our case are viruses or viral strains/haplotypes. Therefore, we included a stage which contains modules to complete a multiple sequence alignment, optimize a model of sequence evolution (Darriba et al. 2020), and generate a maximum likelihood phylogenetic tree. We plan on including more evolutionary analysis programs as modules in a future version of HAPHPIPE. A beginner’s guide to phylogenetic analysis in HAPHPIPE is available in the User Guide (https://gwcbi.github.io/haphpipe_docs/phylo/#phylo-quick-start).

###### Multiple Sequence Alignment (multiple_align)

Prior to generating a phylogeny, a multiple sequence alignment (MSA) needs to be generated in order to infer homologous regions. Because of the ability to include a large number of sequences, quick run time, and high alignment accuracy, we chose to use MAFFT (Multiple Alignment using Fast Fourier Transform) to generate a MSA (Katoh et al. 2002, 2005; Katoh and Toh 2008; Katoh and Standley 2013). Furthermore, MAFFT is open source, maintained, and included as a recipe in Bioconda. The input for this module can be a list of directories which contain all the final.fna files or a FASTA file, or both (in which case the sequences in the FASTA file are combined with the final.fna files retrieved before alignment). If the final.fna contains amplicons or separate MSAs are desired for amplicons, a GTF file can be supplied as well. However, if a GTF file is not supplied, then the option –alignall needs to be specified, and then all input sequences will be aligned together. The outputs of this module located in the subdirectory hp_alignments are alignments in FASTA format; if PHYLIP formatted files (a different format to store an MSA; derived from Felsenstein 2005) are desired, the option –phylipout can be included in the command. PHYLIP or FASTA output may be used to run the build_tree module. Similarly, CLUSTAL format (Thompson et al. 1994; Sievers et al. 2011) can be used by including the –clustalout option. A variety of other options for MAFFT can be found in the User Guide (https://gwcbi.github.io/haphpipe_docs/phylo/#multiple_align). Example to execute: haphpipe muliple_align –dir_list demo_sra_list.txt –ref_gtf HIV_B.K03455.HXB2.gtf –phylipout –logfile demo_multiple_align.log.

###### Model of Evolution Selection Test (model_test)

To select models of evolution, we implement ModelTest-NG (Darriba et al. 2020), which analyzes a multiple sequence alignment and determines the best-fit model of evolution based on statistical criteria (criteria being: AIC—Akaike 1974; AICc—Hurvich and Tsai 1989; BIC—Schwarz 1978; and DT—Minin et al. 2003) as the module model_test. The primary and only required input for this module is an alignment file (either in FASTA or PHYLIP format), which is generated from the previous module muliple_align. As the next module, build_tree, builds a phylogenetic tree using RAxML, an option to output a command with the corresponding evolutionary model can be executed by using the option –template raxml. Partitions (such as different codon positions or gene regions) can be specified as input as well. The outputs of this module are a text file containing the test results and a summary TSV file that contains the input file and the corresponding best-fit evolutionary model for each statistical criterion. An example of both output files are in the User Guide (https://gwcbi.github.io/haphpipe_docs/phylo/#model_test). Example to execute: haphpipe muliple_align –seqs alignment_region00.fasta –template raxml –run_id alignment_region00.

###### Phylogenetic Tree (build_tree)

To estimate a phylogenetic tree, we implement RAxML-NG (Kozlov et al. 2019), an open-source, well-maintained, and efficient phylogenetic analysis tool. RAxML-NG includes a variety of options on reconstructing trees for both nucleotides and amino acids. The input for this module can be either a multiple sequence aligned FASTA file or a PHYLIP formatted file (both can be outputted from the module multiple_align). The number of bootstrap replicates and a partition file are among the common options included as a part of this module. A partition file is often used for denoting different evolutionary models for different genes or codon positions, whereas bootstrap replicates are used to determine the support for nodes within the phylogeny. There are a variety of other options, which are discussed in more detail in the User Guide (https://gwcbi.github.io/haphpipe_docs/phylo/#build_tree_NG). The outputs for this module are located in the subdirectory hp_tree and are formatted specifically for phylogenetic trees. For example, the “raxml.bestTree” file contains the best scoring maximum likelihood tree; the “raxml.boostraps” file contains all trees constructed for bootstrap analysis; the “raxml.support” file contains the best tree with bootstrap support values. This final file (raxml.support) is the tree file that should be used to visualize the tree using visualization programs such as iTOL (Letunic and Bork 2019) or FigTree (https://github.com/rambaut/figtree). Example to execute: haphpipe build_tree –all –seqs alignment_region00.fasta –output_name alignment_ region00.

### Example Pipelines

Two example pipelines have been included in the documentation of HAPHPIPE, both of which are for amplicon assembly (fig. 1): one that implements de novo assembly (haphpipe_assemble_01) and one that uses reference-based mapping (haphpipe_assemble_02).

#### De Novo Assembly

The de novo assembly begins with 1) trimming the raw reads (haphpipe trim_reads), then 2) error-correction (haphpipe ec_reads) using the trimmed reads as input. The trimmed reads are used as input for the 3) de novo assembly step using SPAdes (haphpipe assemble_denovo). The trimmed reads are used here, because SPAdes automatically completes an error correction step during de novo assembly. Hence, we do not want to do error-correction twice on the reads. The assembled contigs (denovo_contigs.fa) are used as input for 4) assembling the amplicons (haphpipe assemble_amplicons), where a reference sequence FASTA file and reference GTF file are also required as inputs. The assembly is then 5) refined (haphpipe refine_assembly) iteratively, with a maximum refinement of five steps, until there is no improvement to the refined sequence. The corrected FASTQ reads are used as input, along with the reference FASTA file this time being the assembled FASTA file (amplicon_assembly.fna). The final step for the de novo assembly pipeline is 6) finalizing the assembly. Here, we used the corrected FASTQ reads again as input with the reference sequence FASTA file being the final refined FASTA file (refined.fna). The final output for a sample is an aligned BAM file (reads relative to final.fna), a FASTA file with the final consensus sequences for the amplicons (final.fna), and a VCF file containing the variants relative to the sample’s consensus sequence (final.fna). Example to execute: haphpipe_assemble_01 SRR8525886_1.fastq SRR8525886_2.fastq HIV_B.K03455.HXB2.fasta HIV_B.K03455. HXB2.gtf PL12.

#### Reference-Based Assembly

The reference-based mapping assembly also begins with 1) trimming the raw reads (haphpipe trim_reads), followed by the trimmed reads then being 2) error-corrected (haphpipe ec_reads). These preliminary cleaning steps are standard practice in the analysis of NGS data. The corrected FASTQ reads are used as input for 3) the reference-based mapping assembly step (haphpipe refine_assembly), with a maximum refinement of five iterative steps, until there is no improvement to the refined sequence. The reference FASTA file contains the reference sequences for the amplicons. The 4) final step is finalizing the assembly, where the corrected FASTQ reads are used again as input with the reference sequence FASTA file being the final refined FASTA file (refined.fna). The final output for a sample is an aligned BAM file (reads relative to the reference amplicon sequences), a FASTA file with the final consensus sequences for the amplicons, and a VCF file containing the variants relative to the reference FASTA file sequences (in the demo, we used HXB2). Example to execute: haphpipe_assemble_02 SRR8525886_1.fastq SRR8525886_2.fastq HIV_B.K03455.HXB2.amplicons.fasta PL12.

The example pipelines are written in bash scripting language. To execute the de novo assembly pipeline on your own data, simply state haphpipe_assemble_01, read_1 file, read_2 file, the reference FASTA file for the assembly, the reference GTF file for your amplicons of choice, and the sample name (see User Guide at https://gwcbi.github.io/haphpipe_docs/expipes/). Alternatively, for the reference-based mapping assembly pipeline, state haphpipe_assemble_02, read_1 file, read_2 file, the reference amplicon FASTA file for the assembly (this can have multiple sequences in the same file), and the sample name. Use the examples given above as a guide. The reference files used in the example pipelines (HIV_B.K03455.HXB2.fasta, HIV_B.K03455.HXB2.gtf, and HIV_B.K03455.HXB2.amplicons.fasta) are included within the demo data when downloaded from GitHub. If you prefer whole-genome assembly and still want to use the pipelines we have included, state the starting position and ending position in your GTF file relative to your virus of choice’s reference sequence FASTA file (i.e., for HIV-1, the numbering would be 1 and 9,719).

### Advanced Users: Creating Your Own Pipeline

Advanced users can easily build their own pipeline using any of the stages described above in any order they choose or even swapping out particular algorithms at particular steps (e.g., alternative haplotype callers, alternative multiple sequence alignment algorithms, alternative phylogenetic estimators, etc.). However, familiarity with general NGS analysis and bash scripting practices are essential to developing your own pipeline. The modular state of HAPHPIPE and the starting directory structure facilitates bash scripting (see the code for the example pipelines) by using the outputs of one module for input into another (see https://gwcbi.github.io/haphpipe_docs/inout/for list of the files output by every module). To run a pipeline on multiple samples with one script, you can easily implement a bash array or loop. Additionally, every module can be called from the command line or within python as a function. The function returns all outputs to the specified output directory, so you can also make a pipeline in python without using bash scripts. Furthermore, a combination of bash and python can be used to construct a “Snakefile” to use Snakemake (Köster and Rahmann 2012), which is a workflow management system. A great starting point for creating your own pipeline is to view the example pipeline codes and adjust each one to your own needs. For example, if you have a large number of reads in your samples, you may want to add a sample_reads step before running de novo assembly. This can be done by adding a sample_reads command to hp_assemble_01 before trim_reads and changing the input files for trim_reads to sample_1.fastq and sample_2.fastq. An example for creating a new custom pipeline for analyzing SARS-CoV-2 can be found in the User Guide (https://gwcbi.github.io/haphpipe_docs/adv/).

## Demo

**Fig. 3.**
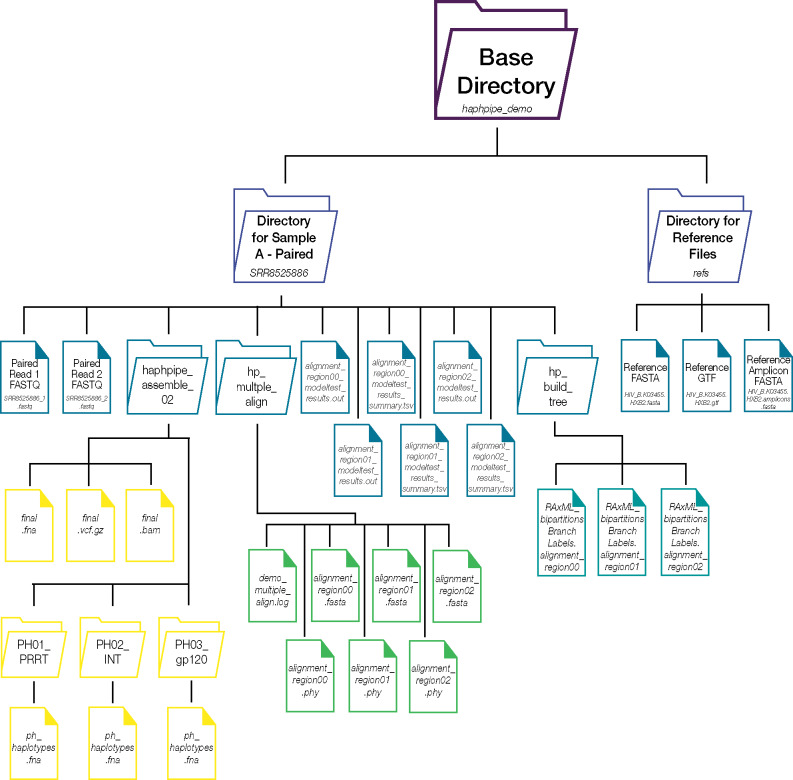
Directory structure after running the demo for one sample. The base directory (haphpipe_demo) should contain one subdirectory for each sample (with a unique and descriptive name) containing sequence data and a separate subdirectory (refs) for the file(s) of viral reference genome(s) and/or genes. The example here shows the structure for the demo data with an example for one sample (SRR8525886). After running the demo, the sample subdirectory will contain three new subdirectories: 1) haphpipe_assemble_02, 2) hp_multiple_align, and 3) hp_build_tree. If PredictHaplo is installed, the haphpipe_assemble_02 subdirectory will contain subdirectories for each region reconstructed. In the case for this demo, there are three amplicon (gene) regions: *PRRT*, *INT*, and *gp120*. A full list of all files present can be found in the User Guide (https://gwcbi.github.io/haphpipe_docs/demos/).

**Fig. 4.**
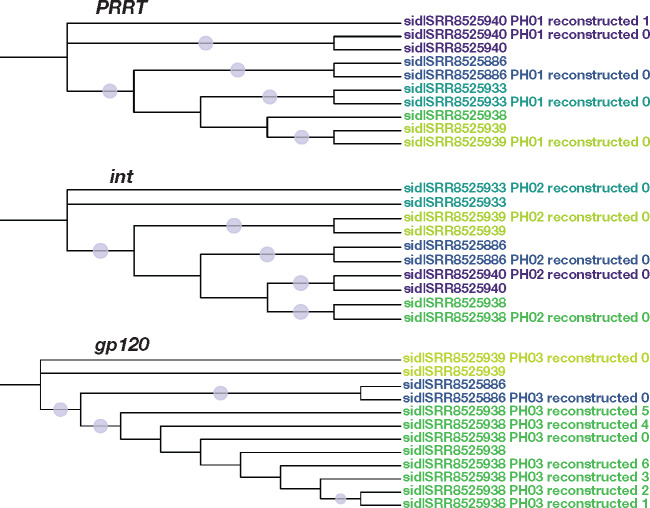
The phylogenetic trees from the competition of the demo. PredictHaplo was installed, so haplotypes were reconstructed for each gene region. Phylogenies for each amplicon genes in the demo (*PRRT*, *int*, and *gp120*) are presented with each sample represented by a different color. Dots correspond to ≥70 bootstrap support.

We provide more details, links, and basic commands for downloading the demo data in the User Guide (https://gwcbi.github.io/haphpipe_docs/demos/). We also describe two strategies to complete the demo: 1) automatically, which is to ultimately test if HAPHPIPE has been installed correctly and 2) interactively, to acquire experience running HAPHPIPE yourself. The demo consists of real HIV-1 data (SRA accessions: SRR8525886, SRR8525933, SRR8525938, SRR8525939, SRR8525940) (Jair et al. 2019) and is processed through haphpipe_assemble_02 amplicon assembly. After running the demo, the files 1) final.fna, 2) final.vcf.gz, and 3) final.bam should be present for each SRA sample within the subdirectory haphpipe_assemble_02 (fig. 3). The FASTA files contain the protease, reverse transcriptase, and *gp120* gene regions. If PredictHaplo is loaded, the haplotype stages will also run, and the files 4) ph_summary.txt and 5) ph_haplotypes.fna for the amplicons should be present within each PH0# subdirectory within each SRA sample directory as well (fig. 3). Finally, the first directory 6) hp_multiple_align should contain the files: demo_multiple_align.log, alignment_region00.fasta, alignment_region00.phy, alignment_region01.fasta, alignment_region01.phy (fig. 3). If PredictHaplo is loaded, alignment_region02.fasta and alignment_region02.phy should also be present. These files for this region (02), which corresponds to *gp120*, is not present when not including the haplotypes, because only three samples are in the alignment and that is too few samples to build a tree in build_tree module with RAxML. Two files 7) alignment_region##_modeltest_results.out and 8) alignment_region##_modeltest_results_summary.tsv should be present for each alignment. The second directory (ix) ph_build_tree should contain the files: RAxML_bipartitionsBranchLabels.alignment_ region00 and RAxML_bipartitionsBranchLabels.alignment_ region01 (fig. 3). Again, if PredictHaplo is loaded and run, then a third file, RAxML_bipartitionsBranchLabels.alignment_ region02, should be present because there were enough samples to build a tree with. Finally, after the demo is completed, the TRE files can be visualized, which we completed with iTOL (fig. 4). The demo runs quickly (around 15–20 min using a single CPU with 16 Gb of RAM) (i.e., available memory). The time speeds up when more CPUs are used, such as when on a high-performance cluster. After a complete run, regardless of if PredictHaplo is installed or not, only 100 Mb of storage is needed. See the User Guide for examples to execute the demo and for more details (https://gwcbi.github.io/haphpipe_docs/demos/).

## Discussion

Here, we introduced HAPHPIPE, a HAplotype and PHylodynamics PIPEline for viral assembly, population genetics, and phylodynamics. This protocol is constructed in a modular fashion such that individual components can be easily replaced with improved methodology or updated versions of the incorporated software. The modular setup facilitates the software’s fluidity to fulfill the needs of the users, who may not need all of the modules or the modules in a particular predefined order to complete their analyses. HAPHPIPE is designed to provide users with an integrated workflow to rapidly analyze viral sequences generated from NGS platforms and provide quality output properly formatted for downstream evolutionary analyses.

To place our software in the context of existing bioinformatics tools, we discuss and compare several alternative viral assembly pipelines all with respect to HAPHPIPE in our validation study (Gibson et al. 2020b). In addition to HIV-1, HAPHPIPE has been tested on other viruses, including HCV and SARS-CoV-2. Each module and both example pipelines were tested on these viruses, and an example of how to build a custom pipeline is included in the User Guide. As most existing viral NGS platforms focus on HIV-1 exclusively, this represents a significant advantage of HAPHPIPE for users analyzing many different viral species. For a thorough comparison of HAPHPIPE’s methods with those of other tools, please see Gibson et al. (2020b). Briefly, we found that HAPHPIPE performed as well or significantly better than each of the other programs tested, while also being able to handle larger amounts of data in a shorter time frame.

We contend that the primary advantages of HAPHPIPE over existing platforms stem from its flexibility, extensibility, and ease of use. HAPHPIPE is open-source and does not contain any requirements or implementation of commercial software or licenses, thus, facilitating its access by a wider community of researchers. In addition, by wrapping multiple programs into convenient and fast pipelines, we greatly simplify the NGS analysis workflow for users at the beginner stage. HAPHPIPE incorporates standardized bioinformatics software through the package manager Conda and the channel Bioconda, which allows for multiple users to access and run HAPHPIPE and greatly simplifies the installation process. Due to the modular system and easily extensible source code, users can customize analyses through a variety of applications and future versions of HAPHPIPE can be readily adapted to include new and updated bioinformatic tools.

Our main goal in writing this protocol is to facilitate the usage of HAPHPIPE to researchers with varying levels of coding experience. Additionally, our commitment to ensuring the public availability of best practices for genomics workflows is carried out through our choice of Bioconda for installing HAPHPIPE. HAPHPIPE provides a user-friendly framework for operating many community-supported open source tools, the underutilization of which, and the resulting implications for rigor and reproducibility in viral genomics, have been brought to light by scientific response to the recent COVID-19 pandemic (Baker et al. 2020). It is our hope that our efforts will further support use, maintenance, and availability of open-source tools within the bioinformatics community.
